# Clinical Spectrum, Radiological Correlation and Outcome of Movement Disorders in Wilson’s Disease

**DOI:** 10.5334/tohm.794

**Published:** 2023-10-09

**Authors:** Rohan R. Mahale, Albert Stezin, Shweta Prasad, Nitish Kamble, Vikram V. Holla, Manjunath Netravathi, Ravi Yadav, Pramod Kumar Pal

**Affiliations:** 1 Department of Neurology, National Institute of Mental Health and Neuro Sciences, Bengaluru, India; 2 Department of Clinical Neurosciences, National Institute of Mental Health and Neuro Sciences, Bengaluru, India

**Keywords:** Wilson’s disease, movement disorders, outcome

## Abstract

**Introduction:**

Movement disorders are the commonest clinical presentation in patients with neurological Wilson’s disease (NWD). There are very few studies evaluating the spectrum, severity and their correlation with magnetic resonance imaging (MRI) changes of movement disorders in NWD.

**Objective:**

To study the spectrum, topographic distribution, radiological correlate, temporal course and outcome in our cohort of NWD patients.

**Methods:**

Retrospective chart review of the NWD patients having movement disorders was performed and analyzed.

**Results:**

Sixty-nine patients (males- 47) with NWD were analysed and the mean age at the onset of neurological symptoms was 13.6 ± 6.6 years (median 13 years; range 7–37 years). The first neurological symptom was movement disorder in 55 (79.7%) patients. Tremor (43.6%) and dystonia (41.8%) was the commonest movement disorder as the first neurological symptom. Dystonia (76.8%) was the most common overall movement disorder followed by parkinsonism (52.1%) and tremors (47.8%). Chorea (10.1%), myoclonus (1.4%) and ataxia (1.4%) were the least common movement disorder. Putamen was the most common affected site (95.6%) followed by caudate nucleus (73.9%), thalamus (60.8%), midbrain (59.4%), internal capsule (49.2%), pons (46.3%). Putamen was the most common area of abnormality in dystonia (98%), tremors (85%). Caudate (75%) and putamen (75%) was the most common areas of abnormality in parkinsonism. Favourable outcome was observed in 42 patients (60.8%) following treatment.

**Conclusion:**

Dystonia is the most common movement disorder in NWD in isolation or in combination with parkinsonism and tremors. Putamen is the most common radiological site of lesions and more frequently affected in patients with dystonia and tremors. Favourable outcome does occur with appropriate medical and surgical treatment.

## Introduction

Wilson’s disease (WD) is an autosomal recessive disorder caused by the genetic mutations in the ATP7B gene resulting in reduced copper (Cu) excretion from the liver causing body copper accumulation primarily in the liver, brain, kidneys and skeletal system [1,2]. The disease was first described in 1912 by Dr Samuel Alexander Kinnier Wilson [3]. Mutation of ATP7B results in the impaired efflux of free Cu to the bile leading to the accumulation of free Cu in the hepatocyte [4]. The transport of copper from the astrocyte to neurons is primarily mediated by ATP7A. The genetic model of WD on the milk mice have shown increased expression of ATP7A in the choroid plexus which results in the neuronal damage due to the increased Cu availability to neurons [5]. The areas of brain which are more susceptible to Cu toxicity due to their higher metabolic rate are the deep gray matter, substantia nigra, and red nuclei [6,7]. The disease has a great phenotypic variability leading to the delay in the diagnosis and effective management [8]. Movement disorders are the commonest clinical presentations in patients with neurological WD (NWD) [9]. The movement disorders in NWD have been classified as (1) an akinetic-rigid syndrome resembling parkinsonism; (2) postural and intention tremor with ataxia, titubation and dysarthria (“pseudosclerosis”); or (3) a generalized dystonic syndrome. Movement disorders do not occur in isolation but as mixed movement disorder [10]. Tremors in WD is frequently postural including rubral (wing-beating), and rest tremors. The action tremor in WD occurs due to copper deposition in the cerebellum [11]. Parkinsonism including rest tremors in WD is due to the nigrostriatal dopaminergic deficits. The response to levodopa is poor due to the damage in the presynaptic and the postsynaptic dopamine receptors [12]. The structural correlate of dystonia in WD is due to the involvement of the basal ganglia-thalamocortical motor circuits [13]. We aimed to study the spectrum, topographic distribution, its radiological correlate, temporal course and outcome in our cohort of NWD patients.

## Subjects and Methods

### Subject recruitment and clinical evaluation

This was a retrospective chart review of patients with WD who were evaluated by the movement disorder specialists at the National Institute of Mental Health and Neurosciences, Bengaluru, India from 2013 to 2021.WD was diagnosed based on the clinical manifestations, evidence of Kayser-Fleischer (KF) rings on slit-lamp examination, low serum copper and ceruloplasmin (< 20 mg/dL) and increased 24-hour urinary excretion of copper (>40 µg) and confirmed using Leipzig criteria for the diagnosis of WD [14]. The phenotypic classification of WD was done using the criteria by Ferenci et al [15]. The NWD was defined as the presence of the neurological and/or psychiatric symptoms at diagnosis. This can be associated with or without symptomatic liver disease. WD patients having movement disorders were included in the study. The case records with incomplete data, without any symptoms of movement disorder were excluded. A detailed review of all the charts was done. The demographic details, age at onset of neurological symptoms, duration of the illness, clinical features with focus on the movement disorders- type, topographic distribution, severity; symptoms of liver dysfunction prior to the neurological symptoms, family history of similar illness, clinical examination findings and investigations including blood investigations, ultrasonography abdomen, brain imaging and genetic data (where available) were collected. The data regarding the brain region involved on MRI were collected by the authors from the reports and scans in the physical and electronic hospital database and were not blinded to the clinical findings. The tremor characteristics were defined according to the revised Consensus criteria for classifying tremor disorders (2018) [16]. The topographic distribution of dystonia was defined according to the Consensus update (2013) [17]. The treatment details including the dose, duration of copper chelators, elemental zinc, paradoxical worsening with copper chelators (if any), medications used for dystonia, tremor, parkinsonism, chorea, myoclonus were recorded. The degree of disability of the patients was assessed using the modified Rankin scale (mRS). The mRS scores were determined at admission and during follow-up based on the details available in the hospital case records. A mRS score 0 represents no symptoms, 1 as no significant disability, 2 as slight disability, 3 as moderate disability, 4 as moderately severe disability, 5 as severe disability and 6 as death. A mRS ≤ 2 was considered as favourable outcome and ≥ 3 as poor outcome [18]. The videos of the patients were reviewed for detailed description of the movement disorder. Institute Ethics Committee approval was obtained for the retrospective analysis of the data (No:NIMH/DO/DEAN (Basic Science)/2020–21). Informed written consent was obtained from the patients for video recording and publication. Patients’ details were anonymized to maintain patient privacy.

#### Statistical analysis

Data was expressed using descriptive statistics. Continuous variables were expressed as mean/median with standard deviation/Inter-quartile range respectively whereas categorical variables were expressed as frequencies and percentages. Statistical analysis was performed using IBM SPSS software version 22.

## Results

A total of 69 patients with NWD were included in the study. The mean age at presentation was 16.3 ± 7.1 years and mean age at onset was 13.6 ± 6.6 years. Forty-seven patients were males (68.1%) and 22 patients were females (31.9%). The median duration of neurological illness was 2 years (Inter-quartile range (IQR)- 1–3 years). The clinical videos were available in our movement disorders database for 15 patients and these were reviewed by the authors in a non-blinded manner. None of the patients had undergone genetic assessment and all patients were diagnosed based on the clinical, radiological and biochemical features.

### Clinical features

Nineteen NWD patients (27.5%) had symptomatic liver disease with history of jaundice before the onset of neurological symptoms. The median age at the onset of symptomatic liver disease in these 19 patients was 6 years (IQR- 2–11 years). Fifty-six patients (81.1%) were born out of consanguineous parentage. Thirteen patients (18.8%) had history of WD in family members mainly in siblings (12 patients), nephew (1 patient). The first neurological symptom was movement disorder in 55 (79.7%) patients. Tremor and dystonia were the commonest movement disorders as the first neurological symptoms. Parkinsonism, chorea and ataxia were the other movement disorder as the first neurological symptoms. Behavioural disturbances (8 patients, 11.6%) and dysarthria (6 patients, 8.7%) were the other first neurological symptom in the remaining 14 patients. Dystonia noted in 53 patients (76.8%) was the most common overall movement disorders followed by parkinsonism (36 patients, 52.1%) and tremors (33 patients, 47.8%). Chorea (6 patients, 10.1%), myoclonus (1 patient, 1.4%) and ataxia (1 patient, 1.4%) were the least common movement disorders. Twenty-nine patients (42%) had single movement disorder during the course of illness. Remaining 40 patients (58%) had combination of movement disorders. Other associated clinical features were dysarthria in 58 (84%), dysphagia in 25 (36.2%), hypersalivation in 50 (72.4%), risus sardonicus in 44 (63.7%), postural instability in 31 (44.9%), falls in 10 (14.5%), behavioural disturbance in 16 (23.2%) and decline in scholastic performance in 24 (34.8%) patients. Table 1 summarises the demographics and spectrum of movement disorders in NWD.

**Table 1:** Demographics and spectrum of movement disorders of patients with NWD.


	NWD PATIENTS (N = 69)

Age at presentation (mean ± SD) years	16.3 ± 7.1

Age at onset (mean ± SD) years	13.6 ± 6.6

Duration of illness (mean ± SD) (range) years	2.02 ± 2.54 (1–14)

Gender (Male: Female)	2:1

NWD without symptomatic liver disease*	50 (72.5)

NWD with symptomatic liver disease*	19 (27.5)

KF ring (%)	69 (100)

Movement disorder as first neurological symptoms*	55 (79.7)

Tremor	24 (43.6)

Dystonia	23 (41.8)

Parkinsonism	6 (10.9)

Chorea	1 (1.8)

Cerebellar ataxia	1 (1.8)

Overall movement disorders*	69 (100)

Dystonia	53 (76.8)

Parkinsonism	36 (52.1)

Tremors	33 (47.8)

Chorea	07 (10.1)

Cerebellar ataxia	01 (1.4)

Myoclonus	01 (1.4)

Combination of movement disorders (n)*	40 (57.9)

Dystonia-parkinsonism	16 (40)

Tremor-dystonia-parkinsonism	12 (30)

Tremor-dystonia	6 (15)

Chorea-dystonia	5 (12.5)

Chorea-dystonia-parkinsonism	1 (2.5)


*Values are expressed as number (percentage); SD-standard deviation. KF ring: Kayser-Fleischer ring; NWD: Neurological Wilson disease; SD: Standard deviation.

#### Spectrum of movement disorders (n = 69)

##### Dystonia (n = 53)

Generalised dystonia was the most common topographical distribution and occurred in 42 patients (79.2%). The onset was limb-onset before generalisation in 40 patients and craniocervical-onset in 2 patients. The median time to generalisation was 1 year (IQR- 0.5–2 years) after onset of dystonia. Two patients with generalized dystonia presented as status dystonicus. The remaining 11 patients (20.7%) with dystonia had segmental topographical distribution (bibrachial dystonia in 7, cervico-brachial dystonia in 2 and bilateral foot dystonia in 2 patients). None of the patients had focal, hemi or multifocal dystonia topography.

##### Tremor (n = 33)

Bibrachial tremors was seen in all 33 patients (100%). The combination of rest tremors with postural and intention tremors of upper limbs occurred in 28 patients (84.8%) and bibrachial postural tremors alone were present in the remaining five patients (15.1%). Wing-beating tremor component was noted in 10 patients (30.3%). No patients had isolated rest tremors of limbs. In addition to bibrachial tremors, eight patients (24.2%) had additional bicrural tremors while five patients (15.1%) had additional jaw tremors.

##### Parkinsonism (n=36), chorea (n = 7) and combination of movement disorders (n = 40)

Of the total 36 patients (52.2%) who had parkinsonism in the form of bradykinesia and rigidity, 34 patients (94.4%) had symmetric parkinsonism while the remaining two (5.6%) had asymmetric parkinsonism. Chorea was observed in seven patients (10.1%) in total and all of them had generalised chorea phenotype. Based on the associated phenomenology, isolated chorea was seen in one patient (14.3%), chorea in combination with dystonia was seen in five patients (71.4%). and chorea with dystonia-parkinsonism in one patient (14.3%).

In total, 40 patients (57.9%) had a combination of two or more movement disorders phenomenology. Dystonia-parkinsonism occurred in 16 patients (40%), tremor-dystonia-parkinsonism in 12 patients (30%), tremor-dystonia in six patients (15%), chorea-dystonia in five patients (12.5%) and chorea-dystonia-parkinsonism in one patient (2.5%). The case-based demonstration of movement disorders is available as clinical vignettes at the end of the results.

##### Laboratory data

The mean serum total copper was 26.9 ± 11.8 mg/dL (range- 10–52 mg/dL), serum ceruloplasmin was 4.5 ± 2.9 mg/dL (range- 1–16 mg/dL) and 24 hours urine copper prior to decoppering treatment was 203.7 ± 87.1 µg/dL (range- 105–188 µg/dL).

##### MRI brain findings (Figures 1, 2)

**Figure 1:**
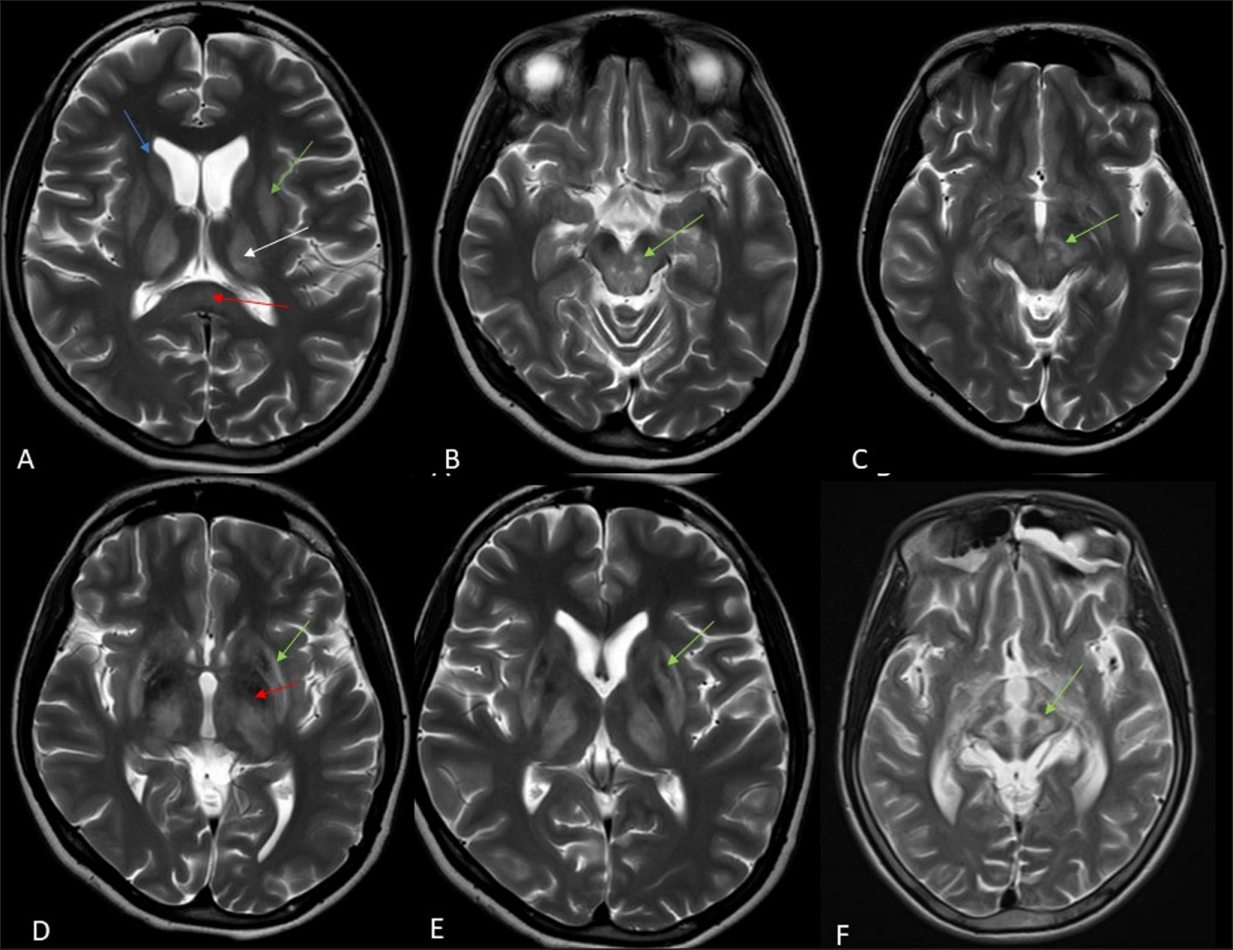
Brain MRI axial T2-weighted images showing hyperintense lesions in **(A)** bilateral caudate (blue arrow), putamen (green arrow), thalamus (white arrow) and splenium of corpus callosum (red arrow); **(B)** and **(C)** midbrain tegmentum (green arrow); **(D)** ‘Bright claustrum’ sign (green arrow), hypointense lesions in bilateral globus pallidum (red arrow); **(E)** central T2-hypointense lesion surrounded by hyperintensity in bilateral putamen (green arrow); **(F)** ‘Face of giant panda’ sign in midbrain (green arrow). MRI: Magnetic resonance imaging.

**Figure 2:**
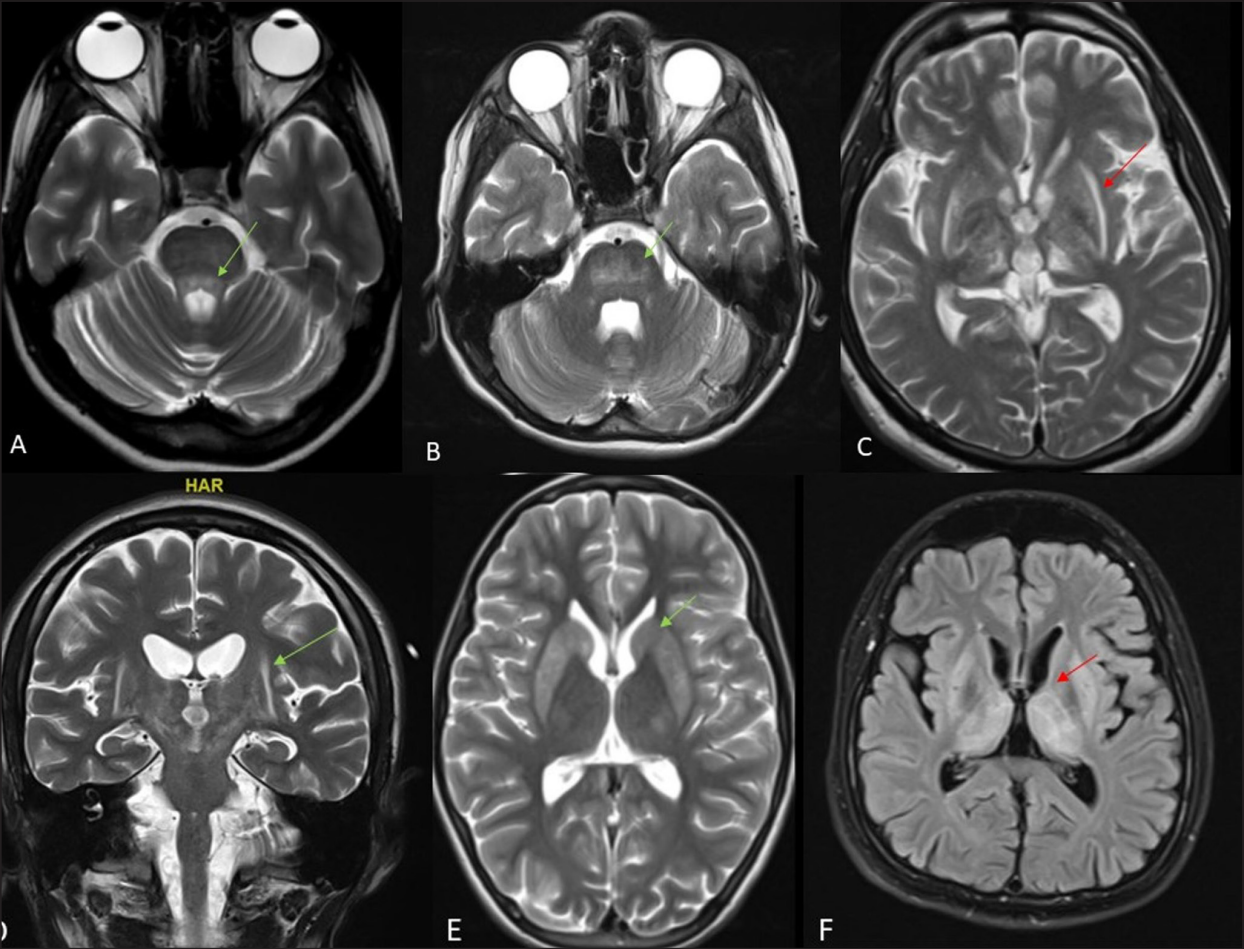
Brain MRI axial T2-weighted images showing hyperintense lesions in **(A)** pontine tegmentum with hypointense central tegmental tract suggesting ‘face of panda cub’ sign (green arrow); **(B)** basis pontis suggesting ‘Trident’ sign (green arrow); **(C)** and **(D)** ‘Bright claustrum’ sign on axial (red arrow) and coronal (green arrow) image; **(E)** bilateral caudate and putamen with sparing of globus pallidus (green arrow); **(F)** Axial fluid-attenuated inversion recovery image showing hyperintense lesion in globus pallidus (red arrow) apart from bilateral caudate, putamen and thalamus.

Brain MRI was done in all patients and abnormalities were noted in all patients. The abnormalities were iso to hypointense lesions on T1-weighted imaging and hyperintense lesions on T2/Fluid-attenuated Inversion Recovery (FLAIR) suggestive of edema, neuronal necrosis or gliosis. Putamen was the most common affected site (95.6%) followed by caudate nucleus (73.9%), thalamus (60.8%), midbrain (59.4%), internal capsule (49.2%), pons (46.3%), cortex (8.7%), globus pallidus (7.2%) and cerebellum (2.9%). “Face of the giant panda” sign was noted in 41 patients (59.4%), “Face of panda cub” in 30 patients (43.4%) and “double panda” sign in 30 patients. Putamen was the most common area of abnormality in dystonia (98%), tremors (85%). Caudate (75%) and putamen (75%) was the most common areas of abnormality in parkinsonism. The frequency of MRI lesions is summarised in Table 2.

**Table 2:** Frequency of MRI lesions in WD patients with dystonia, tremor and parkinsonism.


BRAIN REGION INVOLVED^#^	*DYSTONIA (n = 53)*	*WITHOUT DYSTONIA (n = 16)*

Caudate	41 (77.3)	10 (62.5)

Putamen	**52 (98.1)**	14 (87.5)

Thalamus	35 (66.3)	8 (50)

Midbrain	33 (62.2)	8 (50)

Pons	25 (47.4)	7 (43.7)

Internal capsule	27 (51.1)	6 (37.5)

	***TREMORS (n = 33)***	***WITHOUT TREMORS (n = 36)***

Caudate	19 (57.5)	31 (86.1)

Putamen	**28 (84.8)**	36 (100)

Thalamus	22 (66.7)	20 (55.6)

Midbrain	19 (57.5)	20 (55.6)

Pons	13 (39.3)	17 (47.2)

Internal capsule	9 (27.2)	22 (61.1)

	***PARKINSONISM (n = 36)***	***WITHOUT PARKINSONISM (n = 33)***

Caudate	**27 (75.0)**	24 (73.1)

Putamen	**27 (75.0)**	33 (100)

Thalamus	17 (47.2)	23 (69.8)

Midbrain	20 (55.5)	18 (54.5)

Pons	17 (47.2)	15 (45.4)

Internal capsule	18 (50.0)	17 (51.5)


Values are expressed as number (percentage). The most common region involved in each phenotypic subgroup is marked in bold. ^#^ MRI lesions were T1 iso to hypointense and T2 hyperintense lesions suggestive of edema/necrosis/gliosis/demyelination. MRI: Magnetic resonance imaging; WD: Wilson’s disease.

##### Treatment

Thirteen patients received penicillamine and elemental zinc before the present hospital visit. The median duration of treatment received before the present hospital visit was 2 years. All patients received elemental zinc and penicillamine. The median dose of penicillamine was 500 mg per day (IQR-250–750 mg), median dose of elemental zinc was 150 mg per day. Sixty-three patients received trihexyphenidyl with a mean dose of 8.9 ± 5.2 mg/day (range- 4–24 mg/day). Fifty-six patients received clonazepam with a mean dose of 1.0 ± 0.3 mg/day (range- 0.5–2 mg/day). Levodopa was started for 27 patients with a mean dose of 256.8 ± 91.6 (range- 150–550 mg/day).

##### Surgical treatment

Eight NWD patients (5 males) of this study had undergone surgery for either tremor (unilateral thalamotomy in 4 patients) or dystonia (bilateral GPi-DBS in 2 patients and unilateral pallidotomy in 2 patients). There was significant improvement in Fahn-Tolosa-Marin tremor rating scale (FTM) score from 64, 58, 45 82 to 6, 14, 6, 5 at 10 years, 2 months, 10 years and 2 years follow-up respectively in the 4 patients who underwent thalamotomy for tremor. However, there was no significant improvement either with pallidotomy or with GPi-DBS in those patients with dystonia (The data of these patients have not been included in the present study as they are already published) [19].

##### Outcome (Figure 3)

**Figure 3:**
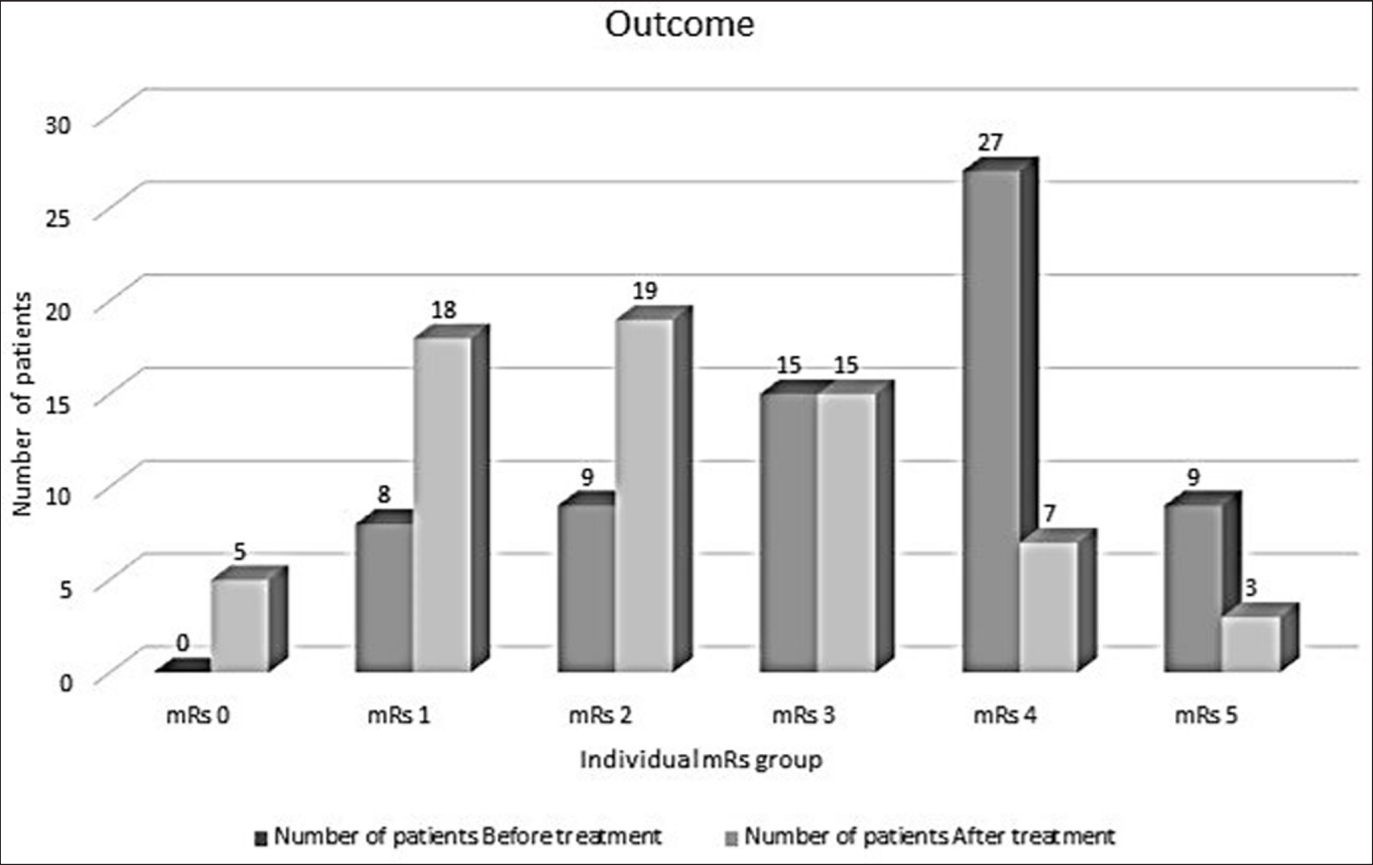
Bar diagram depicting the modified Rankin scale (mRS) outcome of neurological Wilson’s disease before and after treatment. NWD: Neurological Wilson’s disease.

The median mRs score at baseline was 4. The median follow-up of patients was 3 years (IQR-2–5 years). Eighteen patients (26%) had mRs score ≤ 2 at admission and 51 patients (74%) had mRs score ≥ 3. Following treatment, 42 patients (60.8%) had mRs score ≤ 2 and 25 patients had mRs score ≥ 3 (36.2%). Two patients had status dystonicus requiring ventilator support. One patient with status dystonicus had no change in mRs score at 1-year follow-up and the other patient had change in mRs score by 1 point (from 5 to 4 at 2-years follow-up). There were no mortalities during hospital admission. Two patients were lost to follow-up.

#### Case vignettes

##### Case-1

A 33-year-old male presented with tremors of right followed by left upper limbs of 7 years duration. The tremors were present during rest and on maintaining posture with ‘wing-beating’ tremor (Video 1). KF ring was positive. Serum free copper was elevated and serum ceruloplasmin was low. Brain MRI showed signal changes in bilateral caudate, putamen, midbrain and dorsal pons. Patient was started on propranolol 40 mg/day and clonazepam 1 mg/day along with zinc acetate and penicillamine 500 mg/day. He had 40–50% improvement at 6-months follow-up.

**Video 1:** Video of Case-1 showing tremor in both upper and lower limbs (rest, posture with wing-beating quality) along with dystonic posturing of fingers of both hands.

##### Case-2

A 22-year-old male presented with abnormal posturing of both lower limbs while walking for 5 years followed by abnormal posturing in both hands for 3 years, repeated involuntary protrusion of tongue, dysarthria, dysphagia of 1 year duration. The symptoms were progressive. He had positive KF ring, dystonia of both upper and lower limbs with tremors, trombone tongue (Video 2). Brain MRI showed signal changes in bilateral caudate, putamen and thalamus. Patient was started on trihexyphenidyl 12 mg/day and clonazepam 2 mg/day along with zinc acetate and penicillamine 500 mg/day. He had 30% improvement at 6-months follow-up.

**Video 2:** Video of Case-2 showing dystonia of both upper and lower limbs with tremors, trombone tongue.

##### Case-3

A 16-year-old male presented with abnormal posturing of lower limbs, trunk and upper limbs with dysarthria of 3 years duration. The symptoms were progressive and became bed-bound within 3 years of onset. He had positive KF ring, dystonia of both upper and lower limbs, trunk and cranial dystonia with foot contractures (Video 3). Brain MRI showed signal changes in bilateral caudate, putamen, thalamus and midbrain. Patient was started on trihexyphenidyl 12 mg/day, tetrabenazine 100 mg/day, baclofen 40 mg/day and clonazepam 2 mg/day along with zinc acetate and penicillamine 500 mg/day. He had 20% improvement at 6-months follow-up. He had generalized dystonia with fixed posture. Hence, the response to medications was sub-optimal.

**Video 3:** Video of Case-3 showing dystonia of both upper and lower limbs, trunk and cranial dystonia with foot contractures.

##### Case-4

A 19-year-old male presented with abnormal posturing of lower limbs while walking of 3 years duration followed by abnormal involuntary dance-like movement of the body of 2 years duration. On examination, he had choreiform movement of upper limbs, trunk and lower limbs with cervical, trunk and toe dystonia (Video 4). There was no null point or increase in the severity of involuntary movements on walking. Brain MRI showed signal changes in bilateral caudate, putamen and thalamus. Patient was started on tetrabenazine 50 mg/day and clonazepam 1 mg/day along with zinc acetate and penicillamine 500 mg/day. He was lost to follow-up.

**Video 4:** Video of Case-4 showing choreiform movement of upper limbs, trunk and lower limbs with cervical, trunk and toe dystonia.

##### Case-5

A 10-year-old boy presented with abnormal involuntary dance-like movement of the lower limbs, trunk and upper limbs of 1 year duration. Child also had stereotypic movement of right upper limb in the form of repeated flexion of right upper limb. On examination, he had generalised chorea with motor stereotypy of right upper limb and facial dystonia in the form of ‘vacuous smile’ (Video 5). Patient was started on tetrabenazine 25 mg/day and clonazepam 1 mg/day along with zinc acetate and penicillamine 500 mg/day. There was no significant improvement at 6 months follow-up.

**Video 5:** Video of Case-5 showing generalised chorea with motor stereotypy of right upper limb and facial dystonia in the form of ‘vacuous smile’.

## Discussion

**Figure 4:**
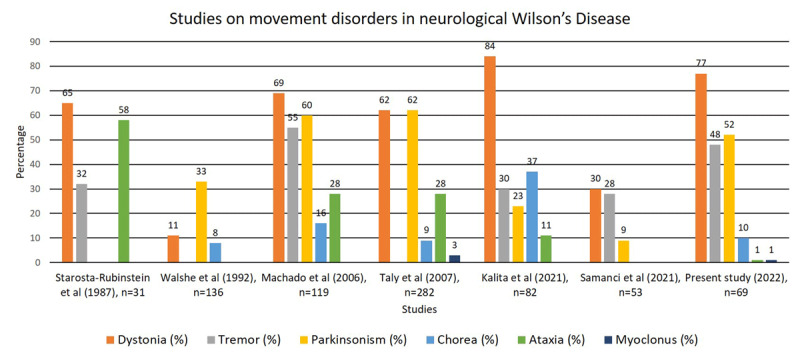
Bar diagram depicting summaries of studies on spectrum of movement disorders in neurological Wilson’s disease.

The aim of this study was to describe the spectrum, topographic distribution, its radiological correlate, temporal course and outcome in our cohort of NWD patients. There are studies which have described the phenomenology of movement disorders in WD. Dystonia was the most common movement disorder in NWD as reported by several studies including the present study (Figure 4). Starosta-Rubinstein et al (1987) studied 31 WD patients and found dystonia as the most common movement disorder constituting about two-thirds of cases. Ataxia, rigidity, gait and postural abnormalities and tremor were the other movement disorders [20]. Similarly, Machado et al (2006), Kalita et al (2021) and Samanci et al (2021) reported dystonia as the commonest movement disorder [21,22,23]. Dystonia was the common movement disorder in our cohort with the limb-onset generalized dystonia as the common topographical distribution. However, two large cohort of WD patients by Walshe et al,1992 and Taly et al, 2007 reported parkinsonism as the commonest movement disorder in NWD followed by dystonia [24,25]. The probable reason may be due to higher frequency of involvement of midbrain in their cohort [24,25]. Parkinsonism was the second common movement disorder in our cohort which was symmetrical. Similarly, parkinsonism was reported by other study as the second common movement disorder [21]. However, two cohorts reported a frequency of 9–23% [22,23]. Nearly half of our patients had postural tremors of upper limbs with wing-beating tremor in one-third of patients with tremor. The frequency of tremors in other studies ranged from 30 to 50% [21,22,23,24,25]. Ataxia, chorea and myoclonus were the least common movement disorders in our cohort. However, Kalita et al (2021) reported chorea as the second common movement disorder [22]. They did not find any correlation of MRI lesions and chorea. The frequency of ataxia in the three cohort were 28–58% but we had a frequency of 1.4% [20,21,25]. Similarly, the frequency of myoclonus was 3–11% [21,25]. Patients with NWD have combination of movement disorders (58%) and it was the most common movement disorder phenomenology in our study. Dystonia-parkinsonism with tremors was the frequent combination of movement disorders. This is summarised in Table 3.

**Table 3:** Summaries of studies on spectrum of movement disorders in NWD.


AUTHOR (YEAR)	STUDY SAMPLE SIZE	STUDY REGION	MOVEMENT DISORDERS SPECTRUM

Starosta-Rubinstein et al (1987)	31	USA	Dystonia (65%) Dysdiadochokinesia (58%) Rigidity (52%) Tremor (32%)

Walshe et al (1992)	136	UK	Parkinsonism -45 (33%) Dystonia- 15 (11%) Chorea- 11 (8%)

Svetel et al (2001)	27	Yugoslavia	Dystonia- 10 (37%)

Machado et al (2006)	119	Brazil	Dystonia (69%) Parkinsonism (60%) Postural tremors (55%) Ataxia (28%) Chorea and Athetosis (16%)

Taly et al (2007)	282	India	Parkinsonism (62.3%) Dystonia (62.3%) Ataxia (28%) Chorea (9%) Myoclonus (3%) & Athetosis (2%)

Kalita et al (2021)	82	India	Dystonia – 69 (84.1%) Chorea- 31 (37.8%) Tremor- 24 (29.3%) Parkinsonism-19 (23.2%) Athetosis-13 (15.9%) Myoclonus-9 (11.0%)

Samanci et al (2021)	53	Turkey	Dystonia- 30% Tremor -28% Parkinsonism – 9%

Present study	69	India	Dystonia (76.8%) Parkinsonism (52.1%) Tremors (47.8%) Chorea (10.1%) Myoclonus (1.4%) Ataxia (1.4%) *Combination of movement disorders* Dystonia-parkinsonism (23.1%) Tremor-dystonia-parkinsonism (17.4%) Tremor-dystonia (8.7%) Chorea-dystonia (7.2%)


NWD: Neurological Wilson’s disease; UK: United Kingdon; USA: United States of America.

Most of the studies have shown frequent lesions in the caudate, putamen, thalamus, midbrain, pons, and subcortical white matter on brain MRI [20,22,25,26]. Less common area of lesions are cerebral cortex, medulla and cerebellum. This is summarized in Table 4. The correlation of each movement disorder with MRI lesions is difficult as there are different types of movement disorder and multiple MRI lesions in each patient. One study found that the dystonia and bradykinesia in NWD correlated with putamen lesions [20,27]. Another study showed that the thalamus and basal ganglia involvement were associated with dystonia and cerebellar lesions with tremors [22]. We found that the putamen was the most common site affected followed by the caudate nucleus, thalamus, midbrain which is similarly reported in the above studies. The involvement of putamen was the most common in patients with dystonia, tremors whereas caudate and putamen involvement was most frequent in parkinsonism presentation. The lesions were iso to hypo on T1 and hyperintense on T2-weighted images suggestive of edema, gliosis, demyelination or neuronal necrosis [13]. Owing to the paucity of data, the anatomical correlate of dystonia, tremor, chorea, parkinsonism in NWD needs further research looking into the network/circuit dysfunctions causing movement disorder.

**Table 4:** Summary of studies on the frequency of brain MRI lesions.


AUTHOR (YEAR)	STUDY SAMPLE SIZE	STUDY REGION	BRAIN MRI LESIONS (%)

Starosta-Rubinstein et al (1987)	31	USA	Caudate (46%), putamen (41%), midbrain (27%), Thalamus (9%), subcortical white mater (23%), pons (23%), Cerebellum (14%), Globus pallidus (5%)

Svetel et al (2001)	27	Yugoslavia	Globus pallidus (48.1%), putamen (44.4%), Claustrum (29.6%), caudate (14.8%), thalamus (18.5%), cerebellum (18.5%), midbrain (14.8%), pons (14.8%)

Sinha et al (2006)	93	India	Putamen (72%), caudate (61%), thalamus (58%), midbrain (49%), pons (20%), cerebral white matter (25%), cortex (9%), medulla (12%) and cerebellum (10%)

Taly et al (2007)	282 MRI (n = 40)	India	Putamen (77.5%), caudate (62.5%), thalamus (60%), globus pallidus (37.5%), midbrain (52.5%), and cerebellum (7.5%)

Kalita et al (2021)	82	India	Thalamus (76.8%), globus pallidus (71.9%), Putamen (69.5%), caudate (68.2%), Brainstem (60.9%), cortex (30.5%), cerebellum (14.6%).

Present study	69	India	Putamen (95.6%), caudate (73.9%), thalamus (60.8%), midbrain (59.4%), internal capsule (49.2%), pons (46.3%), cortex (8.7%), globus pallidus (7.2%) and cerebellum (2.9%)


MRI: Magnetic resonance imaging; USA: United States of America.

Taly et al (2007) reported improvement following decoppering treatment in 78% of their patients and 50% of those patients who had improvement resumed their previous level of activity [25]. Similarly, Linn et al (2009) studied long-term (13 years) exclusive zinc monotherapy in NWD and reported improvement in 9 out of 10 patients with NWD (mRS of 1) [28]. Stanković et al (2023) studied long-term outcome of 40 NWD patients on stable decoppering treatment (mean of 15 years) and found significant decrease in the frequency of dysarthria, clumsiness, tremor, gait disturbance, postural instability and an improvement in school/work performance while dysphagia, drooling, bradykinesia and rigidity, dystonic and choreatic features did not change. Dystonia at disease onset was the only identified predictor of the worse long-term outcome [29]. In our study, the patients were started on chelating agents and symptomatic therapy based on the clinical findings. Surgical interventions such as lesioning or DBS were considered and performed in patients with dystonia and/or tremors which were disabling and sub-optimally responding to anti-dystonic/ anti-tremor medications. The treatment outcome was favourable in 61% of patients following decoppering and symptomatic treatment as compared to the prior treatment proportion of patients with mRs score ≥ 3 (74%). About 30% of patients were asymptomatic or had no significant disability post decoppering treatment. More than one-third of patients, continued to have mRS score of ≥ 3 post-treatment. Most of these patients had generalized dystonia with or without parkinsonism including the two patients with status-dystonicus. Early diagnosis and timely commencement of decoppering treatment will yield favourable outcome in NWD patients.

The strength of the study is the detailed clinical description, radiological correlation and outcome of the movement disorder with treatment. The limitation of this study was the retrospective analysis of clinical, radiological, treatment profile and outcome, lack of objective assessment of frequency and severity of tremors, dystonia severity and lack of genotype data. The re-analysis of brain MRI images by blinding to the clinical findings would have given more insight into the lesional correlation with the clinical manifestations.

## Conclusion

Movement disorder is the most important manifestation of neurological Wilson disease. Among the various movement disorders seen in Wilson’s disease, dystonia was the most frequent, occurring either in isolation or in combination with parkinsonism and tremors. In most patients with dystonia, the distribution was generalized with onset in the limbs, whereas for tremor, it was bibrachial segmental. The combination of postural and intention tremors was more frequent compared to Wing-beating tremors. Parkinsonism was often symmetric whenever it was present. Putamen was the frequent radiological site of lesions and was often associated with dystonia, parkinsonism and tremors. The classical sign of “Face of the giant panda” was absent in one-third of patients. More than two-thirds of the patients had a favourable outcome with one-third of the patients resuming their previous level of activity. Neurological Wilson’s disease has a diverse spectrum of movement disorders and its recognition helps in early diagnosis and effective treatment.
